# Development and Validation of a Sonication-Assisted Dispersive Liquid–Liquid Microextraction Procedure and an HPLC-PDA Method for Quantitative Determination of Zolpidem in Human Plasma and Its Application to Forensic Samples

**DOI:** 10.3390/molecules29112490

**Published:** 2024-05-24

**Authors:** Inés Sánchez-Sellero, Pamela Cabarcos-Fernández, María Elena Jaureguízar-Rodríguez, Iván Álvarez-Freire, María Jesús Tabernero-Duque, Ana María Bermejo-Barrera

**Affiliations:** Forensic Toxicology Service, Forensic Sciences Institute, Faculty of Medicine, Universidade de Santiago de Compostela, C/San Francisco s/n, 15782 Santiago de Compostela, Spain; ines.sanchez.sellero@usc.es (I.S.-S.); mariaelena.jaureguizar@rai.usc.es (M.E.J.-R.); ivan.alvarez@usc.es (I.Á.-F.); mj.tabernero@usc.es (M.J.T.-D.); anamaria.bermejo@usc.es (A.M.B.-B.)

**Keywords:** zolpidem, dispersive liquid–liquid microextraction, HPLC-PDA, quantification, validation, forensic

## Abstract

The use of z-drugs has increased worldwide since its introduction. Although the prescribing patterns of hypnotics differ among countries, zolpidem is the most widely used z-drug in the world. Zolpidem may be involved in poisoning and deaths. A simple and fast HPLC-PDA method was developed and validated. Zolpidem and the internal standard chloramphenicol were extracted from plasma using a sonication-assisted dispersive liquid–liquid microextraction procedure. The method was validated including selectivity, linearity, precision, accuracy, and recovery. The calibration range (0.15–0.6 µg/mL) covers therapeutic and toxic levels of zolpidem in plasma. The limit of quantification was set at 0.15 µg/mL. Intra- and interday accuracy and precision values were lower than 15% at the concentration levels studied. Excellent recovery results were obtained for all concentrations. The proposed method was successfully applied to ten real postmortem plasma samples. In our series, multiple substances (alcohol and/or other drugs) were detected in most cases of death involving zolpidem. Our analytical method is suitable for routine toxicological analysis.

## 1. Introduction

**Figure 1:**
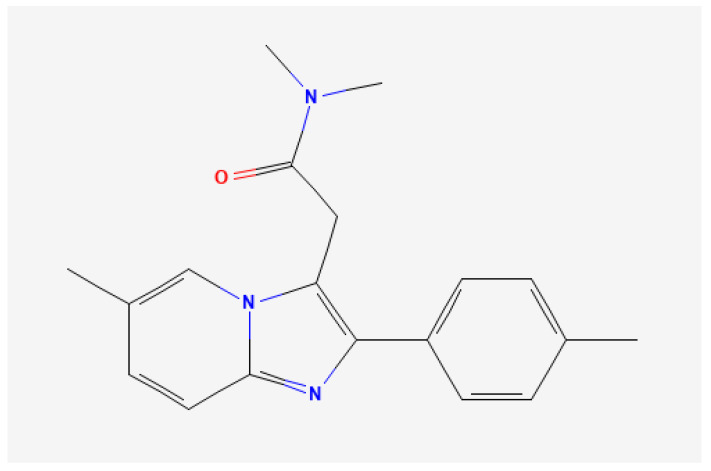
Chemical structure of zolpidem.

Zolpidem, *N,N*-6-trimethyl-2-(4-methylphenyl)imidazo [1,2-α]pyridine-3-acetamide, is an imidazopyridine derivative (Figure 1) [1]. Zolpidem is a nonbenzodiazepine hypnotic indicated for the short-term treatment of insomnia in adults. Zolpidem is rapidly absorbed (20–40 min), showing an oral bioavailability of approximately 70%. The mean time to maximum plasma concentration is 1.4 h [2].

The drug is approximately 90% protein-bound and has a limited volume of distribution. It is extensively metabolized to inactive metabolites by cytochrome P450 enzymes in the liver, predominantly CYP3A4 [3,4]. It has a short elimination half-time (2–3 h). Zolpidem has hypnotic effects by reducing sleep latency and improving sleep quality [4]. It is a γ-aminobutyric acid (GABA)_A_ receptor agonist. Zolpidem preferentially binds to the benzodiazepine ω_1_-receptor, which is thought to be the most related to sleep [3]. Pharmacokinetic and pharmacodynamics interactions between zolpidem and other compounds have been demonstrated. Drug interactions are predictable for compounds such as zolpidem metabolized by CYP3A4. Zolpidem has a CNS-depressant effect, which may be increased by concomitant administration of other CNS-depressant drugs.

The use of z-drugs has increased worldwide since its introduction. The prescribing patterns of hypnotics differ among countries, with zolpidem being the most widely used z-drug in the world. Whilst, in Norway, zopiclone is the dominant z-hypnotic [5,6], zolpidem is by far the most prescribed z-hypnotic drug in Spain with 7.64 defined daily doses (DDDs)/1000 inhabitants/day, whereas zopiclone has much lower prescriptions with 0.54 DDDs in 2021 [7]. As reported by the Spanish Agency for Medicines and Health Products (AEMPS), in Spain, zolpidem is the second most prescribed hypnotic drug (22.42% DDDs of all hypnotic and sedative drugs prescribed in 2021), after lormetazepam (70.57% DDDs of hypnotic and sedative drugs in 2021) [7].

Although zolpidem is usually well tolerated, some adverse effects were reported, and these reactions appear to be dose-related and worse in the elderly. Lower initial doses of zolpidem are recommended in geriatric and/or debilitated patients [3,4,6]. Clinical use of zolpidem is related to some adverse events such as anterograde amnesia or hallucinations [3,8]. Poisoning with zolpidem predominantly involves sedation and coma. Although rare, deaths from zolpidem have been reported, and are more likely to occur with polydrug overdose [4]. Abuse, tolerance, dependence, and withdrawal are reported with z-drugs, though these seem to be less severe and with lower incidence than for benzodiazepines [3,4]. Z-drugs may be involved in drug-facilitated crimes like drug-facilitated sexual assaults. The short elimination time and the delay between crime and clinical examination make toxicological analysis of these drugs difficult [9].

Several analytical methods have been reported for the quantification of zolpidem in blood, including HPLC with fluorescence detection [2,10], HPLC-DAD [11], GC/MS [12], GC/MS/MS [13], LC/MS [8,14], UPLC/MS [15], LC/MS/MS [16,17,18,19,20,21], and UHPLC-MS/MS [5,22,23]. The most commonly used techniques for zolpidem extraction from biological matrices are liquid–liquid extraction (LLE) [2,5,14,16,19,20,21] and solid-phase extraction (SPE) [8,12,15]. Although SPE is a popular extraction technique, it shows several disadvantages such as long extraction times, complex multi-step procedures, and expensive columns. LLE consists of faster and less complex steps compared to SPE; however, it consumes large volumes of volatile organic solvents. In accordance with the principles of Green Chemistry, there are new trends in sample extraction procedures such as microextractions, defined by the small volumes of solvents used. Microextraction by packed sorbent (MEPS), a miniaturized version of SPE, and dispersive liquid–liquid microextraction (DLLME) were recently applied to extract z-hypnotics from plasma [23] and whole blood [17], respectively. DLLME is one of the most interesting alternative solvent-minimizing procedures for sample preparation applied in the field of forensic toxicology.

The aim of this study was to develop a fast and simple HPLC-PDA method for the quantitative determination of zolpidem in human plasma covering a high therapeutic/toxic range. DLLME was optimized as a new extraction procedure, which is faster and with lower organic solvent expense than traditional SPE and LLE techniques. The validation of the analytical method was carried out according to the guidelines of the Food and Drug Administration (FDA) [24]. The limit of detection (LOD) of this validated method was 0.05 µg/mL and the limit of quantification (LOQ) was 0.15 µg/mL. The developed method was successfully applied to ten human plasma samples received at the Forensic Toxicology Service of the Forensic Sciences Institute of Santiago de Compostela (Spain).

## 2. Results

### 2.1. Study of Extraction Procedure

The extraction efficiency in DLLME can be improved by the optimization of certain experimental variables, including the type and volume of extraction and disperser solvents, the sample volume, the pH of the aqueous phase, the ultrasound time, and the amount of salt added.

#### 2.1.1. Study of the Type and Volume of Extraction and Disperser Solvents

One of the most important factors is the selection of the type and volume of the extraction solvent. The most frequently used are chlorinated solvents. The extraction solvent must be immiscible with water and miscible with the disperser solvent, and must be able to dissolve the analyte of interest. Maximum extraction efficiencies are usually observed at low extraction volumes (20–200 µL). Extraction solvent needs to have a higher density than water so that, after centrifugation, it would form the lower phase in the conical tube [25,26].

Another important factor is the type and volume of disperser solvent. It has to be miscible with both the extraction solvent and the aqueous sample. The most frequently used disperser solvents are acetonitrile and methanol. A few hundred microliters (200–1000 µL) are often enough to disperse the organic extraction solvent in the sample. Lower volumes of disperser solvent may not be sufficient, and therefore, the cloudy solution may not be properly formed. On the other hand, larger volumes of disperser solvent are discouraged due to the cosolvent effect which decreases the extraction efficiency [25,26].

In this study, combinations of different extraction solvents (i.e., chloroform, carbon tetrachloride, dichloromethane, ionic liquid) and several disperser solvents (i.e., methanol, acetonitrile, acetone) were tested. The mixture containing carbon tetrachloride (as the extraction solvent) and acetonitrile (as the disperser solvent) appeared to be the best for forming droplets.

#### 2.1.2. Study of Buffer and pH

Taking into account that the pK_a_ value of zolpidem is 6.2, a pH range of 6 to 12 was tested using different buffers. Phosphate buffer at pH 6, phosphate buffer at pH 7.5, ammonium buffer at pH 8, ammonium buffer at pH 12, borate buffer (pH 9), and sodium carbonate buffer (pH 9.5) were evaluated in duplicate. It was found that the best results were obtained with the sodium carbonate buffer at pH 9.5.

#### 2.1.3. Study of Several Variables with Statistical Package

To select the best conditions for DLLME, a total of 18 experiments were performed with the following factors: extraction solvent volume, disperser solvent volume, buffer volume, and sample volume (Table S1). The experimental design was constructed using StatGraphics Centurion statistical package (18.1.12 version; Statgraphics Technologies, Inc., The Plains, VA, USA). Different ranges of each variable were tested (extraction solvent volume: 40–150 µL; disperser solvent volume: 0.5–1.5 mL; buffer volume: 0.2–1 mL; sample volume: 0.5–1 mL).

**Figure 2:**
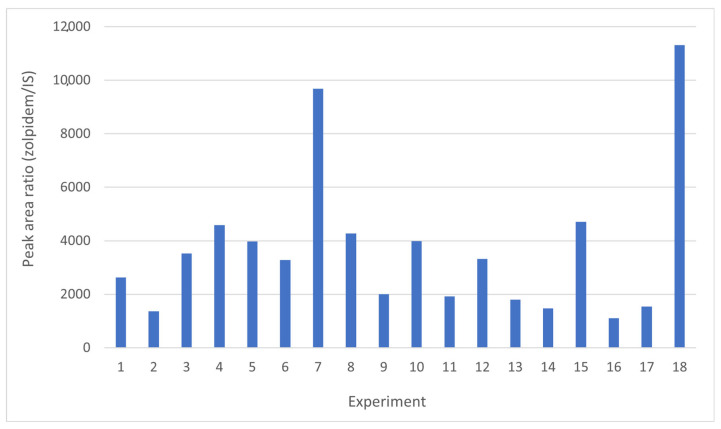
Results obtained from the 18 experiments performed in order to study the influence of sample volume, buffer volume, extraction solvent volume, and disperser solvent volume following the conditions shown in Table S1.

**Figure 3:**
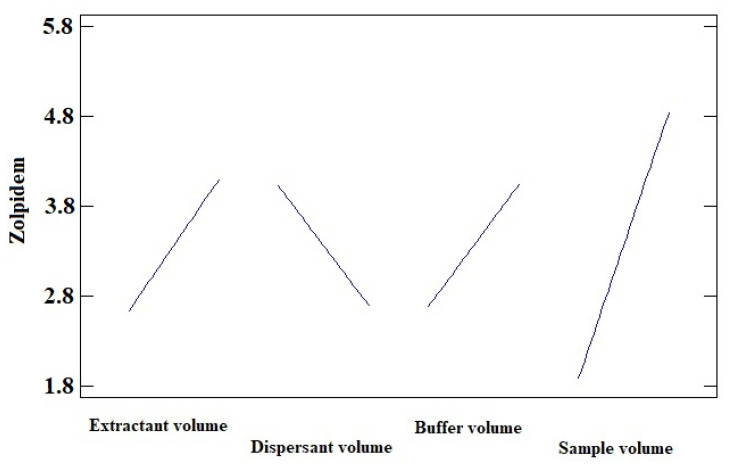
Main Effects Plot for zolpidem (sodium carbonate buffer pH 9.5, acetonitrile as disperser solvent, and CCl_4_ as extraction solvent).

Figure 2 graphically shows the peak area ratio of Zolpidem/IS achieved in each of the 18 experiments carried out combining these four variables. Once this information was entered into the statistical program StatGraphics, the Main Effects Plot was obtained (Figure 3), where the variability of each of the factors studied can be observed.

Figure 3 shows that as the volume of the extractant and dispersant solvent decreases, the recovery of the analyte under study increases. However, recovery is enhanced by increasing the volume of the sample and buffer solution.

Finally, the optimal conditions provided by the statistical program were as follows: 0.75 mL of plasma were diluted with 0.6 mL of sodium carbonate buffer (pH 9.5), and 60 µL of carbon tetrachloride and 0.5 mL of acetonitrile were used as the extraction and disperser solvents, respectively.

#### 2.1.4. Study of Salt Amount

Different amounts of sodium chloride (0.5, 1, 2, 5, and 10% *w*/*v*) were tested in duplicate. The addition of 0.5% *w*/*v* salt provided the best results.

#### 2.1.5. Study of Sonication

Sonication can be used to assist the dispersion of the extraction solvent in the aqueous phase in order to reduce the extraction time. A sonication time range from 5 min to 25 min was tested and compared with no sonication. Sonication improved extraction recoveries compared with no sonication, and 5 min of sonication was chosen as the best condition tested.

### 2.2. Choice of Internal Standard

Chloramphenicol was successfully tested and used as the internal standard (IS) in our method.

### 2.3. Chromatographic Separation

**Figure 4:**
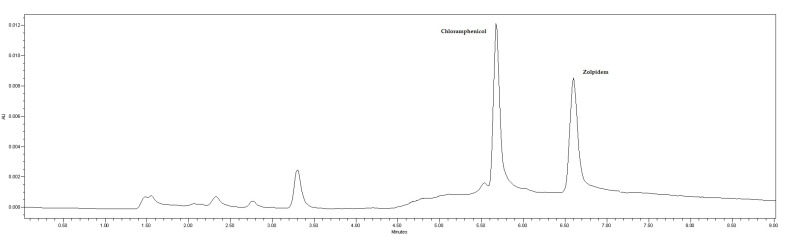
Chromatogram of a standard solution of chloramphenicol (IS) and zolpidem. Retention times of 5.67 and 6.57 min for IS and zolpidem, respectively.

As mentioned in the Materials and Methods section, the mobile phase was a mixture of 1 mM phosphate buffer (pH 8) and acetonitrile pumped at a flow rate of 1 mL/min. A gradient elution program was applied, starting at 26% acetonitrile and keeping constant for 0.5 min. Afterwards, a linear gradient reaching 55% acetonitrile in 2 min was established, followed by a linear decrease, back to 26% acetonitrile in 6.5 min. The injection volume was 20 µL and the run time was 9 min. Chromatographic separation was carried out on a reversed-phase column. These chromatographic conditions provided optimal separation of IS and zolpidem, with retention times (t_R_) of 5.67 min and 6.57 min, respectively (Figure 4).

**Figure 5:**
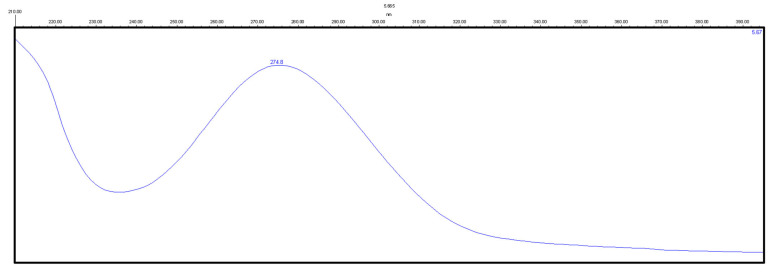
Absorbance spectrum of a standard solution of chloramphenicol.

**Figure 6:**
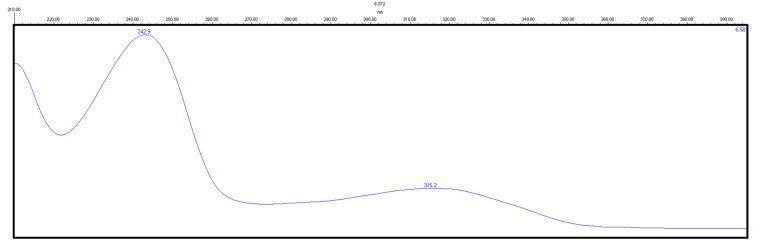
Absorbance spectrum of a standard solution of zolpidem.

Detection was monitored at 275 and 243 nm for chloramphenicol (Figure 5) and zolpidem (Figure 6), respectively.

### 2.4. Method Validation

#### 2.4.1. Selectivity

Blank plasma samples from six different untreated individuals spiked with IS were analyzed. No interfering peaks from endogenous substances were found at the retention times of zolpidem and IS (Figure S1).

#### 2.4.2. Linearity

Seven calibration curves were constructed using blank plasma samples spiked with zolpidem to obtain seven calibrators from 0.15 to 0.6 µg/mL. All standard samples were spiked with 20 µL of IS (100 µg/mL chloramphenicol). Calibration curves were obtained on different days. A linear least-squares regression model was applied and the results obtained showed a consistent correlation between the zolpidem/IS peak area ratios and the corresponding nominal concentration of zolpidem, with a correlation coefficient >0.99.

#### 2.4.3. Limits of Detection and Quantification

The LOD, defined as the lowest concentration giving a signal-to-noise ratio of three, was 0.05 µg/mL. The LOQ, calculated at a signal-to-noise ratio of five, was set at 0.15 µg/mL.

#### 2.4.4. Precision and Accuracy

The intraday and interday precision and accuracy results obtained in human plasma at three concentration levels (quality controls QC_1_ = 0.15 µg/mL, QC_2_ = 0.4 µg/mL, and QC_3_ = 0.6 µg/mL) are shown in Table 1. The intra- and interday precision values (% RSD) did not exceed 9.36%, and the intra- and interday accuracy values (% *bias*) ranged from 0.84 to 14.97%. All precision and accuracy data were within the acceptance criteria defined by the FDA [24].

#### 2.4.5. Recovery

Recoveries of zolpidem obtained in low-, medium-, and high-quality control samples (0.15, 0.4, and 0.6 µg/mL, respectively) are shown in Table 1. Recoveries were within a range of 100.84 and 121.01%.

#### 2.4.6. Method Application

**Figure 7:**
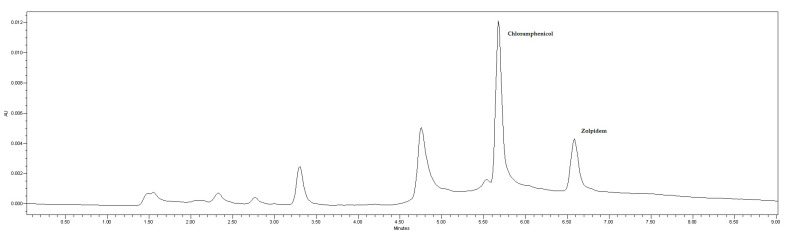
Chromatogram of a postmortem sample received at the Forensic Toxicology Service of the Forensic Sciences Institute of Santiago de Compostela (Spain) and analyzed by the developed and validated method.

The proposed method was applied to ten human postmortem samples sent to our laboratory for analysis. Table 2 shows the results obtained in terms of zolpidem concentration in human plasma and additional information such as sex, age, and the cause of death suspected by a medical examiner based on the history and autopsy. Table 2 also lists other drugs identified and quantified in blood, as well as drugs detected in other samples such as urine, gastric contents, or vitreous humor. Zolpidem concentrations quantified ranged from 0.18 µg/mL to 0.33 µg/mL. The chromatogram of one of these real postmortem samples is shown in Figure 7.

## 3. Discussion

Zolpidem is a widely prescribed hypnotic for the short-term treatment of insomnia in adults. It has a short elimination half-time. Pharmacokinetic and pharmacodynamics interactions between zolpidem and other compounds have been reported. Drug interactions are predictable for compounds metabolized by CYP3A4. Zolpidem has a CNS-depressant effect, which may be increased by concomitant administration of other CNS-depressant drugs. Zolpidem may be associated with adverse and dangerous effects even if it is used at therapeutic doses. Although rare, deaths from zolpidem have been reported, and are more likely to occur with polydrug overdose [4].

Several analytical methods have been reported for the quantification of zolpidem in blood. Many of them are excellent and sensitive methods, with LOQs lower than ours, due to the use of more sensitive instrumental techniques. However, such equipment is not always available for routine analysis in many laboratories. We developed a fast and simple HPLC-PDA method for the quantitative determination of zolpidem in plasma. The calibration range of 0.15–0.6 µg/mL covers therapeutic and toxic levels of zolpidem in plasma samples. The developed method was validated according to the guidelines of the FDA [24]. The LOD was 0.05 µg/mL and the LOQ 0.15 µg/mL.

The most commonly used techniques for zolpidem extraction from biological matrices are liquid–liquid extraction (LLE) [2,5,14,16,19,20,21] and solid-phase extraction (SPE) [8,12,15]. These extraction procedures suffered from some disadvantages such as long extraction times, multiple phases, expensive columns, or large volumes of organic solvents. In accordance with the principles of Green Chemistry, there are new trends in sample extraction procedures such as microextractions. DLLME is one of the most interesting alternative solvent-minimization sample preparation procedures. It can provide less consumption of organic solvents and higher recoveries due to the large contact surface area of the extraction solvent. There are some publications in the scientific literature that employ DLLME to determine drugs in blood samples, such as De Boeck et al. [17] and Fisichella et al. [18]. The first author used this technique to extract benzodiazepine-like hypnotics from whole blood samples using an ionic liquid, while the second author used this microextraction technique for the analysis of several drugs, including Zolpidem, and drugs of abuse. In general, DLLME is a scarcely used procedure for the extraction of zolpidem. In our study, different variables of DLLME were optimized (type and volume of solvents, buffer volume, and sample volume). DLLME was demonstrated as a satisfactory extraction procedure for zolpidem, faster and with lower organic solvent consumption than traditional methods such as SPE and LLE.

The developed method was successfully applied to ten human plasma samples received at the Forensic Toxicology Service of the Forensic Sciences Institute of Santiago de Compostela (Spain). In eight cases, zolpidem was suspected to be involved. In five of these cases, zolpidem had been medically prescribed (cases 1, 5, 7, 8, and 10). In the other three cases (cases 2, 3, and 6), empty packages of medications had been found next to the body at the death scene. In two cases (cases 4 and 9), zolpidem was not initially suspected to be involved. In the first case (case 1), the zolpidem concentration in plasma was within the therapeutic range and the cause of death was natural (urinary tract infection). The cause of death in case 7 was traumatic brain injury and showed therapeutic levels of zolpidem and mirtazapine. The cause of death in case 10 was railway suicide in a man with prescribed psychotropic medication. In this case, zolpidem and sertraline were detected at therapeutic levels. In our series, the cause of death was determined to be “drug poisoning suicide” in 50% of cases (cases 2, 3, 4, 6, and 9) and the zolpidem concentration in plasma ranged from 0.25 µg/mL to >ULOQ, except for case 9 in which zolpidem was detected in gastric contents but not in plasma. In all these fatal poisonings, multiple medications had caused death. In three cases (3, 6, and 9) ethyl alcohol was also involved. A review of the literature suggests that zolpidem alone is rarely the cause of death [3], and multiple substances (alcohol and/or other drugs) were involved in most cases of fatal poisoning [3,27], as was also observed in our study. Zolpidem has a CNS-depressant effect, which may be increased by concomitant administration of other CNS-depressant drugs. Pharmacokinetic and pharmacodynamic interactions between zolpidem and other compounds may occur and have been previously reported.

## 4. Materials and Methods

### 4.1. Chemicals and Reagents

Zolpidem (1.0 mg/mL in methanol) was purchased from Cerilliant^®^ (Round Rock, TX, USA) and the internal standard chloramphenicol (purity ≥ 98%) was supplied by Sigma-Aldrich (St. Louis, MO, USA). HPLC-grade methanol was obtained from Sigma-Aldrich (St. Louis, MO, USA). Acetonitrile was LC-MS-grade (Millipore, Darmstadt, Germany). Carbon tetrachloride was obtained from Merck (Madrid, Spain) and sodium chloride from Panreac (Barcelona, Spain). Anhydrous sodium carbonate (Na_2_CO_3_) and sodium hydrogen carbonate (NaHCO_3_) were analytical-grade and obtained from Panreac (Barcelona, Spain) and Merck (Darmstadt, Germany), respectively. Di-potassium hydrogen orthophosphate trihydrate (K_2_HPO_4_.3H_2_O, purity ≥ 99%) and potassium dihydrogen orthophosphate (KH_2_PO_4_, purity ≥ 99%) were analytical-grade and purchased from Merck (Darmstadt, Germany). HPLC-grade water from the MilliQ-A10 system (Millipore, Bedford, MA, USA) was used.

### 4.2. Blood Samples

Drug-free plasma samples were obtained from the Blood Bank of Santiago de Compostela and used for method validation. Postmortem blood samples received at our Forensic Toxicology Service were stored at –20 °C until use. Plasma was obtained from whole blood by centrifugation (14,000 rpm for 5 min) and kept at 4 °C.

### 4.3. Standard Solutions

Standard solutions of zolpidem of 10 and 100 µg/mL were prepared by dissolving zolpidem 1.0 mg/mL in HPLC-grade methanol. An IS stock solution of 1 mg/mL was prepared by dissolving the pure compound in HPLC-grade methanol. From this stock solution, a 100 µg/mL standard solution of chloramphenicol was obtained by dilution. All stock and standard solutions were stored at –20 °C until use. Standard solutions of zolpidem were used to spike aliquots of blank human plasma to obtain the calibration standard solutions at concentrations of 0.15, 0.2, 0.25, 0.3, 0.4, 0.5, and 0.6 µg/mL of zolpidem. Seven independent calibration samples were prepared and analyzed on separate days. Quality control (QC) samples of zolpidem at three concentration levels, namely low-level QC (QC_1_ = 0.15 µg/mL), medium-level QC (QC_2_ = 0.4 µg/mL), and high-level QC (QC_3_ = 0.6 µg/mL), were also freshly and independently prepared in five replicates in blank plasma.

### 4.4. Sample Preparation and Extraction

In order to optimize the DLLME sample preparation procedure, parameters that could influence extraction efficiencies were assessed. The assessed parameters were the type and volume of disperser solvent (0.5, 1.0, and 1.5 mL), the type and volume of extraction solvent (40, 60, 95, and 150 µL), the type and volume of buffer added (0.2, 0.6, and 1.0 mL), the volume of the sample (0.5, 0.75, and 1.0 mL), the salt effect (0.5, 1, 2, 5, and 10% *w*/*v* NaCl), and the time of sonication (5, 10, 15, 20, and 25 min). The final conditions of the optimized DLLME procedure were as follows: 0.75 mL of plasma was transferred into a conical-bottom glass tube and spiked with 20 µL of standard solution of chloramphenicol (100 µg/mL; internal standard); 0.6 mL of sodium carbonate buffer (pH 9.5) and 0.009 g of sodium chloride (0.5% *w*/*v*) were added. In this procedure, the appropriate mixture of extraction solvent (60 µL carbon tetrachloride) and disperser solvent (0.5 mL acetonitrile) was rapidly injected into the sample solution using a syringe, and then the mixture was gently shaken for several seconds. A cloudy solution of very fine droplets of CCl_4_ dispersed into the sample solution was formed, enabling the transfer of analyte toward the CCl_4_ phase. After sonication for 5 min and centrifugation for 4 min at 4300 rpm, the droplet formed was collected by a 100 µL syringe and transferred to a conical-bottom glass tube. The separated phase was evaporated to dryness at 40 °C under a gentle flow of nitrogen. The residue was then reconstituted with 100 µL of the mobile phase, vortexed, and transferred to an autosampler vial for HPLC-PDA analysis.

### 4.5. Instrumentation and Chromatographic Conditions

The chromatographic analysis was carried out using an HPLC system (Waters 2695 Separations Module) coupled with a PDA (Waters^®^ 996 Photodiode Array Detector) (Waters, Milford, MA, USA). Instrumental parts were controlled, and data processed by Empower^®^ 2 software (Build 2154 Waters). The chromatographic separation was carried out at room temperature on a reversed-phase XBridge^®^ Shield RP18 column (4.6 × 250 mm i.d., 5 µm particle size).

The mobile phase was a mixture of 1 mM phosphate buffer (pH adjusted to 8) and acetonitrile pumped at a flow rate of 1 mL/min. A gradient elution program was set up, starting at 26% acetonitrile and keeping constant for 0.5 min. This condition was followed by a linear gradient reaching 55% acetonitrile in 2 min, followed by a linear decrease, back to 26% acetonitrile in 6.5 min. The injection volume was 20 µL and the run time was 9 min. Detection was monitored at 243 and 275 nm for zolpidem and chloramphenicol, respectively.

### 4.6. Method Validation

The validation of the analytical method was performed following the guidelines established by the FDA [24] in terms of selectivity, linearity, limits of detection (LOD) and quantification (LOQ), intra- and interday precision, accuracy, and recovery.

Selectivity was assessed by analyzing six blank plasma samples from different untreated individuals.

Linearity was evaluated by constructing seven calibration curves at seven concentration levels, including the LOQ and ULOQ (0.15–0.6 µg/mL). Calibration curves were obtained on different days. A linear least-squares regression model was applied based on zolpidem-to-IS peak area ratios.

The LOD was calculated at a signal-to-noise ratio of 3 (S/N = 3). The LOQ, defined as the lowest concentration of the calibration curve that can be measured with acceptable intra- and interday precision and accuracy, was calculated at a signal-to-noise ratio of 5 (S/N = 5).

The precision of the proposed method was established by determining the relative standard deviation (% RSD = 100 × SD/average). Precision was assessed using quality control (QC) plasma samples spiked with zolpidem to obtain three different concentration levels (low QC_1_ 0.15 µg/mL, medium QC_2_ 0.4 µg/mL, and high-level QC_3_ 0.6 µg/mL), within the linear range of the calibration curve of the analyte. Five replicates of each QC sample were analyzed on the same day (intraday precision), and five replicates of each QC sample were analyzed on five different days (interday precision). Accuracy was calculated through relative error (RE, % *bias*), following the same schedule as precision. According to the acceptance criteria, error of accuracy and precision should not exceed 15% for each calibration standards, except at the LOQ, where 20% error is accepted.

Recovery was studied at three concentration levels (0.15, 0.4, and 0.6 µg/mL) in five replicates. Recovery was calculated by comparison of the analytical results obtained when the analyte was added before extraction with those obtained.

## 5. Conclusions

A fast and simple HPLC-PDA method for the quantitative determination of zolpidem in plasma was developed and validated. We optimized sonication-assisted DLLME as an alternative extraction procedure to those that are more conventional. The proposed DLLME was demonstrated as a good microextraction procedure for zolpidem in plasma samples, being faster and using lower volumes of organic solvents than the previously reported traditional SPE and LLE procedures. The method validation, carried out according to the FDA guidelines, showed good linearity, precision, accuracy, and recovery. The calibration range (0.15–0.6 µg/mL) covers therapeutic and toxic levels of zolpidem in plasma samples. The LOD was 0.05 µg/mL and the LOQ was 0.15 µg/mL. The proposed method was successfully applied to ten real postmortem cases and can be used for routine toxicological analysis. According to our results, all deaths involving zolpidem were attributed to other causes different to “zolpidem overdose”. In 50% of cases, the cause of death was “drug poisoning suicide” involving multiple drugs (ethyl alcohol and/or psychotropic medications). In the other cases, toxicological analysis showed therapeutic levels of zolpidem and the cause of death was not related to zolpidem.

## Figures and Tables

**Table 1:** Intraday and interday precision (% RSD), accuracy (% *bias*), and recovery values for zolpidem in human plasma at low (QC_1_), medium (QC_2_), and high (QC_3_) concentrations (n = 5).

C_nominal_^a^ (μg/mL)	Intraday				Interday		
	Precision (% RSD ^b^)	Accuracy (% *Bias*)	Recovery (%)		Precision (% RSD)	Accuracy (% *Bias*)	Recovery (%)
QC_1_ 0.15	8.18	12.69	121.01		8.83	5.29	105.29
QC_2_ 0.40	9.36	14.92	114.92		7.80	0.84	100.84
QC_3_ 0.60	7.55	14.97	116.86		1.96	1.46	101.46

^a^ Nominal concentration; ^b^ relative standard deviation.

**Table 2:** Results of analysis of postmortem plasma samples from forensic cases and additional information.

Real Cases	Sex	Age (Years)	Cause of Death	Zolpidem Concentration in Plasma (μg/mL)	Other Compounds Detected in Blood (Concentration)	Other Compounds Detected (Sample)
1	Male	55	Natural	0.18	─	Trimethoprim (urine)
2	Female	23	Drug poisoning suicide	>ULOQ	Quetiapine (2.3 µg/mL) Topiramate Flurazepam	Quetiapine, topiramate, flurazepam and zolpidem (urine and gastric contents)
3	Female	76	Drug poisoning suicide	>ULOQ	Ethyl alcohol (1.22 g/L) Citalopram (0.03 µg/mL) Trazodone (0.99 µg/mL) Lorazepam (0.14 µg/mL)	Ethyl alcohol, benzodiazepines, citalopram, codeine, trazodone, venlafaxine and zolpidem (urine)
4	Male	47	Drug poisoning suicide	0.33	Alprazolam (0.14 µg/mL) Venlafaxine (>ULOQ) Benzoylecgonine (0.04 µg/mL)	Cocaine, benzodiazepines, venlafaxine, THC and zolpidem (urine)
5	Male	51	Cocaine overdose	<LOQ	Cocaine (4.93 µg/mL) Benzoylecgonine (5.27 µg/mL)	Cocaine, benzodiazepines and ethylglucuronide (urine)
6	Female	60	Drug poisoning suicide	0.25	Ethyl alcohol (0.08 g/L) Quetiapine (2.97 µg/mL) Clozapine (4.8 µg/mL)	Quetiapine, clozapine and zolpidem (urine)
7	Male	81	Traumatic brain injury	0.19	Mirtazapine (0.075 µg/mL)	─
8	Male	38	Drug overdose	<LOQ	Ethyl alcohol (2.47 g/L) Methadone (0.41 µg/mL) Cocaine (0.11 µg/mL) Benzoylecgonine (0.9 µg/mL) Cocaethylene (0.16 µg/mL)	Ethyl alcohol, cocaine, methadone, chlormethiazole and zolpidem (urine)
9	Male	50	Drug poisoning suicide	None detected	Ethyl alcohol (1.72 g/L) Tramadol (0.74 µg/mL) Pregabalin (1.12 µg/mL) Citalopram (0.04 µg/mL) Gabapentin (25.97 µg/mL)	Ethyl alcohol (vitreous humor). Tramadol, pregabalin, citalopram, gabapentin and zolpidem (gastric contents)
10	Male	77	Railway suicide	<LOQ	Sertraline (0.29 µg/mL)	─

## Data Availability

Data is not available due to privacy.

## Supplementary Materials

The following supporting information can be downloaded at https://www.mdpi.com/article/10.3390/molecules29112490/s1: Table S1: Combinations of DLLME parameters studied.
