# Supplementary material for: Development and Validation of a Sonication-Assisted Dispersive Liquid–Liquid Microextraction Procedure and an HPLC-PDA Method for Quantitative Determination of Zolpidem in Human Plasma and Its Application to Forensic Samples
Source: Molecules. 2024 May 24;29(11):2490. doi: 10.3390/molecules29112490 (PMC11173445; doi:10.3390/molecules29112490)
Supplement: Supplementary file 1 [file molecules-29-02490-s001.zip › molecules-3001549-supplementary.pdf]

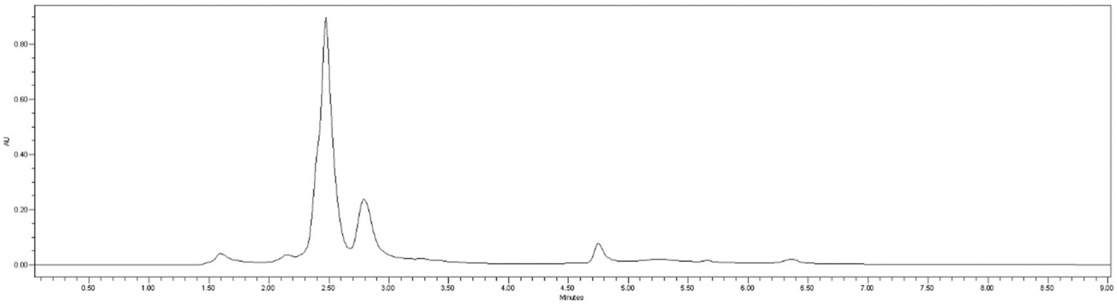

Figure S1. Chromatogram of a blank plasma sample

Table S1. Combinations of DLLME parameters studied.

	Sample volume (mL)	Buffer volume (mL)	Extraction solvent volume (µL)	Disperser solvent volume (mL)
Experiment 1	0.75	0.6	95	1.0
Experiment 2	0.50	0.2	40	0.5
Experiment 3	1.00	0.2	40	0.5
Experiment 4	1.00	0.2	150	1.5
Experiment 5	1.00	1.0	40	1.5
Experiment 6	1.00	1.0	150	1.5
Experiment 7	1.00	1.0	150	0.5
Experiment 8	0.50	1.0	150	0.5
Experiment 9	0.50	1.0	150	1.5
Experiment 10	1.00	1.0	40	0.5
Experiment 11	0.50	1.0	40	1.5
Experiment 12	1.00	0.2	40	1.5
Experiment 13	0.50	0.2	40	1.5
Experiment 14	0.50	0.2	150	0.5
Experiment 15	1.00	0.2	150	0.5
Experiment 16	0.50	0.2	150	1.5
Experiment 17	0.50	1.0	40	0.5
Experiment 18	0.75	0.6	60	0.5
